# Transgenic inhibition of astroglial NF-κB protects from optic nerve damage and retinal ganglion cell loss in experimental optic neuritis

**DOI:** 10.1186/1742-2094-9-213

**Published:** 2012-09-10

**Authors:** Roberta Brambilla, Galina Dvoriantchikova, David Barakat, Dmitry Ivanov, John R Bethea, Valery I Shestopalov

**Affiliations:** 1 Department of Neurological Surgery, The Miami Project to Cure Paralysis, Miller School of Medicine, University of Miami, Miami, FL, 33136, USA; 2 Department of Ophthalmology, Bascom Palmer Eye Institute, Miller School of Medicine, University of Miami, Miami, FL, 33136, USA; 3 Vavilov Institute of General Genetics, Russian Academy of Sciences, Moscow, Russian Federation; 4 Department of Microbiology and Immunology, Miller School of Medicine, University of Miami, Miami, FL, 33136, USA; 5 Department of Cell Biology and Anatomy, Miller School of Medicine, University of Miami, Miami, FL, 33136, USA

**Keywords:** Optic neuritis, Astrogliosis, Retinal ganglion cell death, NF-κB pathway

## Abstract

**Background:**

Optic neuritis is an acute, demyelinating neuropathy of the optic nerve often representing the first appreciable symptom of multiple sclerosis. Wallerian degeneration of irreversibly damaged optic nerve axons leads to death of retinal ganglion cells, which is the cause of permanent visual impairment. Although the specific mechanisms responsible for triggering these events are unknown, it has been suggested that a key pathological factor is the activation of immune-inflammatory processes secondary to leukocyte infiltration. However, to date, there is no conclusive evidence to support such a causal role for infiltrating peripheral immune cells in the etiopathology of optic neuritis.

**Methods:**

To dissect the contribution of the peripheral immune-inflammatory response versus the CNS-specific inflammatory response in the development of optic neuritis, we analyzed optic nerve and retinal ganglion cells pathology in wild-type and GFAP-IκBα-dn transgenic mice, where NF-κB is selectively inactivated in astrocytes, following induction of EAE.

**Results:**

We found that, in wild-type mice, axonal demyelination in the optic nerve occurred as early as 8 days post induction of EAE, prior to the earliest signs of leukocyte infiltration (20 days post induction). On the contrary, GFAP-IκBα-dn mice were significantly protected and showed a nearly complete prevention of axonal demyelination, as well as a drastic attenuation in retinal ganglion cell death. This correlated with a decrease in the expression of pro-inflammatory cytokines, chemokines, adhesion molecules, as well as a prevention of NAD(P)H oxidase subunit upregulation.

**Conclusions:**

Our results provide evidence that astrocytes, not infiltrating immune cells, play a key role in the development of optic neuritis and that astrocyte-mediated neurotoxicity is dependent on activation of a transcriptional program regulated by NF-κB. Hence, interventions targeting the NF-κB transcription factor in astroglia may be of therapeutic value in the treatment of optic neuritis associated with multiple sclerosis.

## Introduction

Optic neuritis (ON) is the earliest manifestation of multiple sclerosis (MS) occurring in approximately 20% of patients [1]. It is characterized by unilateral, painful loss of vision in the absence of neurological or systemic symptoms [1]. Hallmark of the pathology is axonal injury associated with demyelination, which leads to Wallerian degeneration of the optic nerve and, ultimately, delayed death of retinal ganglion cells (RGCs) [2,3]. Although the specific mechanisms responsible for triggering axonal injury and demyelination in ON are unknown, it has been suggested that a key event is the activation of immune-inflammatory processes secondary to leukocyte infiltration into the optic nerve [4-6]. Indeed, studies in mice induced with experimental autoimmune encephalomyelitis (EAE), a model of MS, have shown that ON is accompanied by presence of macrophages [7] and T cells [8] in the optic nerve, and strategies aimed at preventing the recruitment of such immune cells can reduce axonal and myelin damage [7,9]. Nevertheless, there is no conclusive evidence to support a causal role for infiltrating peripheral immune cells in the etiopathology of ON, since no exhaustive studies have been conducted to profile and compare the exact timing of axonal damage and immune cell infiltration in the optic nerve. A mounting body of evidence has underscored that axonal injury in MS and EAE can occur early on in the disease process, even prior to the appearance of clinical symptoms and in the absence of inflammatory infiltrates into the brain or spinal cord [10,11]. Therefore, it is plausible that axonal damage in the optic nerve could also initiate early and independently of immune cell activation. Along with this concept, Howell and colleagues demonstrated that, both in MS (in normal appearing white matter) and EAE, the disruption of the axon/oligodendrocyte association at nodal and paranodal domains is associated with axonal damage and it occurs in parallel with local microglia activation before the infiltration of immune cells [10]. Microglia activation has been shown to occur as early as 7 days after induction of EAE in spinal cord and brain parenchyma, well ahead of leukocyte infiltration [12]. This suggests that resident cells of the central nervous system (CNS) which have immune-inflammatory competence, namely microglia, may play an active role in the early development and progression of axonal injury in MS and ON. In addition to microglia, it has been shown that astrocytic responses contribute to EAE pathology, similarly to other neurodegenerative conditions. Indeed, evidence of axonal injury has been reported in the pre-clinical phase of EAE prior to measurable T cell entry into the CNS parenchyma and in coincidence with astrocytic activation [13]. In our own work we demonstrated that by inhibiting the transcription factor NF-κB selectively in astrocytes with a genetic approach (GFAP-IκBα-dn mice) mice are protected from MOG-induced EAE. This was due to the suppression of astrocyte-dependent inflammation, providing a direct demonstration of the crucial role of astrocytes in the development and progression of the disease [14]. In order to dissect the contribution of CNS-specific versus peripheral immune-inflammatory response in ON, in the present study we compared optic nerve and RGC pathology in wild-type (WT) and GFAP-IκBα-dn mice induced with EAE. We found that, in WT mice, significant axonal and myelin damage in the optic nerve occurred as early as 8 days post induction (dpi) of EAE, prior to even the earliest signs of leukocyte infiltration, that became evident only at 11 dpi (only a few sparse cells) in our experimental paradigm. GFAP-IκBα-dn mice, however, were significantly protected and showed a nearly complete prevention of axonal demyelination, as well as a drastic attenuation of RGC death. This correlated with a decrease in the expression of pro-inflammatory cytokines, chemokines, adhesion molecules, as well as suppression of NAD(P)H oxidase (Cybb/NOX2 and Ncf1 subunits). Combined, these data suggest that NF-κB-dependent events activated in astrocytes in the early stages of disease are key determinants in the initiation and progression of ON.

## Materials and methods

### Mice

Experiments were performed according to protocols approved by the Institutional Animal Care and Use Committee of the University of Miami. GFAP-IκBα dominant negative (GFAP-IκBα-dn) mice were generated in the Transgenic Core Facility of the University of Miami as previously described [15], and extensively characterized under physiological conditions and in various models of neurodegenerative diseases [14,16-22]. GFAP-IκBα-dn mice are designed to expresses a truncated form of the inhibitor of κB alpha (IκBα) under the control of the glial fibrillary acidic protein (GFAP) promoter, resulting in functional inactivation of the NF-κB classical pathway in astrocytes and in GFAP-expressing non-myelinating Schwann cells [15,18,20]. All behavioral, biochemical, and immunological analyses of GFAP-IκBα-dn mice have been previously published [14-18,20]. All mice used in the present study were 2- to 4-month-old females obtained by breeding heterozygous GFAP-IκBα-dn males with WT females. WT littermates were used as controls. Animals were housed in a 12-h light/dark cycle in a virus/antigen free facility with controlled temperature and humidity and provided with water and food *ad libitum*.

### Induction of EAE

Active EAE was induced with MOG_(35–55)_ peptide as previously described [23]. Clinical signs of EAE were assessed daily using a standard scale of 0 to 6 as follows: 0, no clinical signs; 1, loss of tail tone; 2, flaccid tail; 3, complete hind limb paralysis; 4, complete forelimb paralysis; 5, moribund; 6, dead. Mice were considered at onset of EAE when they reached a score of 2 or more for at least 2 consecutive days.

### Total RNA isolation and real-time PCR

After perfusion of the mice with cold PBS, optic nerves were quickly dissected out, homogenized in lysis buffer, frozen instantly and stored in liquid nitrogen until further processing. Prior to RNA extraction, optic nerves were subjected to 20 freeze-thaw cycles. Total RNA was extracted with a resin spin-column system (Absolutely RNA Nanoprep Kit, Stratagene) and reverse transcribed to synthesize cDNA (Reverse Transcription System, Promega). Real-time PCR was performed in the Rotor-Gene 6000 Cycler (Corbett Research) using SYBR GREEN PCR MasterMix (Qiagen) and gene-specific primers (Table 1). Relative expression was calculated by comparison with a standard curve after normalization to the housekeeping gene β-actin.

**Table 1:** Primers for real-time PCR amplification

**Gene**	**Oligonucleotides**		**PCR product size**
IL-1β	Forward	GACCTTCCAGGATGAGGACA	283 bp
	Reverse	AGGCCACAGGTATTTTGTCG	
CCL5	Forward	AGCAGCAAGTGCTCCAATCT	280 bp
	Reverse	ATTTCTTGGGTTTGCTGTGC	
CXCL10	Forward	GCTGCAACTGCATCCATATC	293 bp
	Reverse	CACTGGGTAAAGGGGAGTGA	
ICAM-1	Forward	TGGTGATGCTCAGGTATCCA	273 bp
	Reverse	CACACTCTCCGGAAACGAAT	
TNF	Forward	CAAAATTCGAGTGACAAGCCTG	113 bp
	Reverse	GAGATCCATGCCGTTGGC	
NOS2	Forward	CAGAGGACCCAGAGACAAGC	299 bp
	Reverse	TGCTGAAACATTTCCTGTGC	
Cybb/NOX2	Forward	GACTGCGGAGAGTTTGGAAG	258 bp
	Reverse	ACTGTCCCACCTCCATCTTG	
Ncf1	Forward	CGAGAAGAGTTCGGGAACAG	286 bp
	Reverse	AGCCATCCAGGAGCTTATGA	
β-actin	Forward	CACCCTGTGCTGCTCACC	327 bp
	Reverse	GCACGATTTCCCTCTCAG	

### Immunohistochemistry

Mice were transcardially perfused with 4% paraformaldehyde in PBS. Eyes were enucleated and the optic nerves removed. After washing in PBS, optic nerves were embedded in OCT medium, cryosectioned at a 10 μm thickness and stored at −20°C. Prior to immunostaining, sections were thawed at room temperature, post-fixed for 15 min in 4% paraformaldehyde in PBS, washed and permeabilized in 0.3% Triton X-100 in PBS for 30 min. Tissues were blocked with PBS containing 0.15% Tween 20, 2% bovine serum albumin (BSA), and 5% serum (either goat or donkey to match the secondary antibody) for 30 min at room temperature. Sections were then incubated with primary antibodies (rabbit anti-GFAP, 1:1,000, Dako; rat anti-CD45, 1:250, Invitrogen; goat anti-p47^phox^, 1:200, Santa Cruz) in blocking solution, overnight at 4°C. After thorough washing in PBS, sections were incubated with species-specific fluorescent secondary antibodies for 1.5 h at room temperature. Control sections were incubated with secondary antibody alone. Finally, sections were cover-slipped with Vectashield (Vector) fluorescent mounting medium containing DAPI. Imaging was performed with a Leica TSL AOBS SP5 confocal microscope (Leica Microsystems).

### P-phenylenediamine (PPD) staining of the optic nerve and quantification of myelinated axons

Mice were transcardially perfused with 4% paraformaldehyde in 0.1 M phosphate buffer (pH 7.4). Eyes were enucleated, optic nerves removed and post-fixed in cold 4% paraformaldehyde/2% glutaraldehyde in 0.1 M phosphate buffer overnight, followed by 2% osmium tetroxide in cacodylate buffer (pH 7.4). Tissues were then dehydrated in alcohol and embedded in epoxy resin. Semi-thin sections (1 μm thick) were obtained with a Leica Ultracut E microtome, stained with 1% PPD in methanol-isopropanol (1:1) for 20 min, rinsed with isopropanol and cover-slipped. Samples were then examined by light microscopy on a Zeiss Axioscope and the number of PPD-stained myelinated axons was estimated using the software Stereoinvestigator (MicroBrightfield). For this assessment, six animals/group were used. The analysis was performed at the Image Analysis Core Facility of the Miami Project to Cure Paralysis.

### NeuN immunohistochemistry in flat mounted retinas and quantification of RGC numbers

Eyes were enucleated upon euthanasia, incised at the ora serrata, and immersion-fixed in 4% paraformaldehyde in PBS (pH 7.4) for 1 h. Retinas were then removed and cryoprotected overnight in 30% sucrose. Prior to staining, retinas were subjected to three freeze-thaw cycles, rinsed three times in PBS, and blocked for 1 h in 0.1 M Tris buffer (TB) containing 5% donkey serum and 0.1% Triton X-100. Retinas were then incubated overnight with monoclonal FITC-conjugated NeuN antibody (Chemicon; dilution 1:300). After three rinses in TB, retinas were flat-mounted, cover-slipped and imaged using a Leica TSL AOBS SP5 confocal microscope (Leica Microsystems) equipped with Leica LAS AF software. Quantification of NeuN-positive RGCs was carried out as follows. To avoid topological irregularities, stacks of five serial images were collapsed to generate ‘maximum projections’ (standard feature of the Leica LAS AF software), where all imaged cells appear in sharp focus. Individual retinas were sampled randomly to collect a total of 20 images located at the same eccentricity (1 to 1.5 mm from the optic disk) in the four retinal quadrants using a 20× objective. NeuN-positive neurons with the size range of 6 to 30 μm were counted semi-automatically using MetaMorph (Universal Imaging Co.) software, after image thresholding and manual exclusion of artifacts.

### Statistical analysis

Statistical analysis of EAE clinical scores was carried out with the Mann–Whitney test. Real-time PCR and cell density data were analyzed with one-way ANOVA followed by Tukey test for multiple comparisons. For single comparisons, Student’s t test was applied. *P* values ≤0.05 were considered statistically significant.

## Results

### Inhibition of astroglial NF-κB protects the optic nerve from loss of myelinated axons following experimental ON

**Figure 1:**
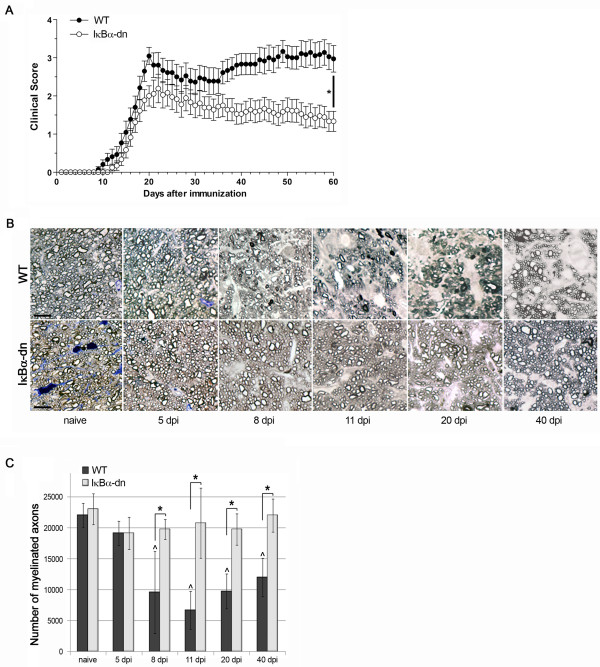
**Histological analysis and quantification of myelinated axons in the optic nerve of mice with ON. (A) **Clinical course of MOG_(35–55)_-induced EAE in WT and GFAP-IκBα-dn mice. EAE symptoms were scored daily for 60 days as described in Materials and Methods. Results are expressed as the daily mean clinical score ± SEM of 24 animals/group from three independent experiments;*P < 0.0001, Mann–Whitney test. **(B)** Histological analysis of PPD-stained, resin-embedded optic nerves of WT and GFAP-IκBα-dn mice under naïve conditions and at various times after induction of EAE. Scale bar = 10 μm. **(C)** Quantification of the total number of myelinated axons in PPD-stained cross-sections of the optic nerve using Stereoinvestigator software; ^*P* < 0.05 *vs.* WT naïve; *P < 0.05, one-way ANOVA, Tukey test. Ten mice/group were analyzed.

In our previous studies with mice genetically modified to suppress NF-κB activation specifically in astrocytes (GFAP-IκBα-dn mice) we demonstrated that astrocyte-dependent inflammatory cascades are key pathological contributors to the development and progression of CNS trauma and disease, including EAE [14,15,17-19,22]. GFAP-IκBα-dn mice exposed to MOG-induced EAE exhibited a significant functional recovery compared to WT mice and this directly correlated with the suppression of pro-inflammatory mediators (chemokines, cytokines, adhesion molecules) in the CNS [14]. Since ON is an early pathological feature of MS and is typically observed in mice induced with EAE, we sought to determine whether astrocyte-dependent responses were involved in the pathophysiology of ON. We subjected GFAP-IκBα-dn mice and WT littermates to MOG-induced EAE as previously described [14], and assessed myelin and axon damage in the optic nerve by counting the number of PPD-stained myelinated axons at various times post induction of the disease. GFAP-IκBα-dn mice steadily recovered from EAE, contrary to WT mice who exhibited chronic exacerbation of the symptoms (Figure 1A), in agreement with our previously published study [14]. In WT mice, loss of myelinated axons in the optic nerve began as early as 5 days after induction of EAE (Figure 1B, C), and reached statistical significance by 8 dpi (Figure 1B, C), before the clinical motor symptoms of EAE became evident (Figure 1A). The peak of axonal demyelination (about 68% reduction of the corresponding naïve condition) was assessed at 11 dpi (Figure 1B, C), when only minimal motor deficits were recorded (clinical score: 0.33 ± 0.16; Figure 1A). No further loss was detected at later time points, either acute (20 dpi) or chronic (40 dpi). In striking contrast to WT mice, GFAP-IκBα-dn mice did not exhibit any reduction in the number of myelinated axons compared to naïve mice at all time points (Figure 1B, C), showing complete protection. From 8 to 40 dpi, the numbers of myelinated axons were consistently and significantly reduced in WT mice compared to transgenics, demonstrating that astrocyte-dependent cellular events are key pathological determinants of the early stages of ON.

### Immune cell infiltration in the optic nerve occurs after onset of axonal demyelination and is not affected by inactivation of astroglial NF-κB

**Figure 2:**
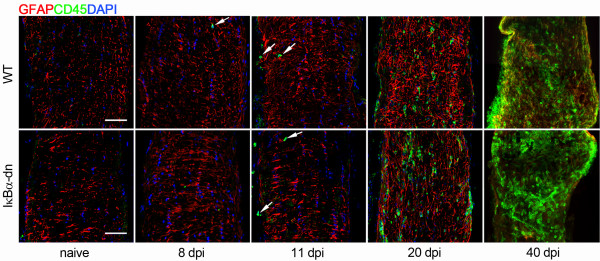
**Leukocyte infiltration in the optic nerve of mice with ON.** Double immunohistochemistry for the astrocyte-specific marker GFAP (red) and the pan-leukocyte marker CD45 (green) in 10-μm thick cryostat-cut sections of the optic nerve of WT and GFAP-IκBα-dn mice under naïve conditions and at various times after induction of EAE. Nuclei were labeled with DAPI. White arrows: isolated CD45^+^ leukocytes. Scale bar = 20 μm.

Since it has been suggested that axonal injury in ON is due to the damaging effects of peripheral immune cells infiltrating into the optic nerve, we set out to investigate whether the protection from axonal demyelination in GFAP-IκBα-dn mice was dependent upon inhibition or delay of immune cell infiltration. Leukocyte infiltration was assessed by anti-CD45 immunohistochemistry on longitudinal sections of the optic nerve collected at various time points after induction of EAE (Figure 2). Interestingly, we detected an identical pattern of infiltration in WT and GFAP-IκBα-dn mice. Indeed, CD45^+^ cells were not present at 5 dpi in both WT and GFAP-IκBα-dn mice (data not shown), and virtually absent at 8 and 11 dpi, where only very few isolated CD45^+^ leukocytes were observed in both genotypes (Figure 2). The first appreciable numbers of infiltrating immune cells were detected at 20 dpi (Figure 2), corresponding to the peak of EAE motor symptoms (Figure 1A). Massive infiltration was evident at the chronic time point of 40 dpi in both WT and GFAP-IκBα-dn mice (Figure 2). These data convey two important points. First, peripheral immune cell infiltration temporally occurs when axonal demyelination in the optic nerve has already taken place. Indeed, in WT mice a significant and robust reduction in the number of myelinated axons is detected at 8 dpi (Figure 1B, C), when essentially no infiltrating cells are present (Figure 2). Second, GFAP-IκBα-dn mice are protected from axonal demyelination long term (40 dpi, Figure 1B, C) despite the massive presence of infiltrating leukocytes at later time points (40 dpi, Figure 2). Taken together these data demonstrate, for the first time, that optic nerve demyelination is an early event in the pathophysiology of ON and it is not secondary to immune cell infiltration and activation. Instead, the early demyelination is dependent upon detrimental cascades initiated by resident CNS cells, namely the astrocytes.

### Inhibition of astroglial NF-κB suppresses the pro-inflammatory response in the optic nerve following experimental ON

**Figure 3:**
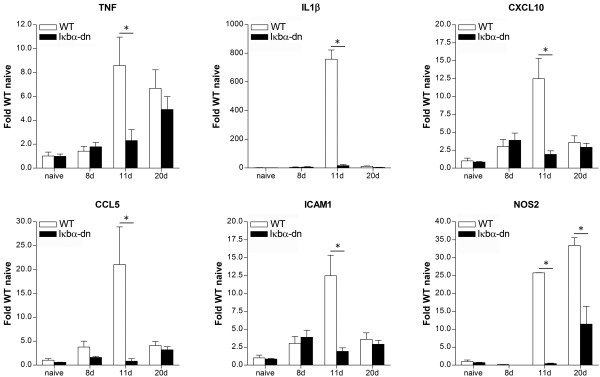
**Pro-inflammatory gene expression in the optic nerve of mice with ON.** Gene expression was assessed in naïve animals and at 8, 11, and 20 dpi. For each gene, results were expressed as fold of corresponding WT naive ± SEM after normalization to β-actin. Four animals/group/time were analyzed. *P < 0.05, one-way ANOVA, Tukey test.

We previously demonstrated that astrocyte-driven inflammation is a key determinant of EAE pathophysiology. Indeed, inhibition of the master regulator of inflammation NF-κB specifically in astrocytes in our GFAP-IκBα-dn mice resulted in significant protection from EAE through the suppression of pro-inflammatory gene expression in the spinal cord and brain [14]. Having demonstrated that GFAP-IκBα-dn mice induced with EAE are also protected from optic nerve demyelination since the early stages of ON (Figure 1), independently of any effect on peripheral immune cell infiltration (Figure 2), we sought to determine whether this could be correlated with a reduction in the expression of pro-inflammatory mediators in the optic nerve, similarly to what we observed in the other CNS compartments previously investigated (spinal cord, brain) [14]. We analyzed gene expression in the optic nerve in naïve animals and at various time points after induction of EAE, focusing on classical pro-inflammatory cytokines and chemokines (TNF, IL1β, CXCL10, CCL5), adhesion molecules (ICAM-1), and the nitric oxide signaling pathway (NOS2). In WT mice we observed that the peak of gene expression occurred at 11 dpi (Figure 3), coinciding temporally with the peak of axonal demyelination (Figure 1C). Importantly, inhibition of astroglial NF-κB in GFAP-IκBα-dn mice resulted in a robust and significant suppression of gene expression compared to WT mice. Specifically, upregulation of IL1β, CXCL10, CCL5, and ICAM-1 were completely prevented. As for TNF, upregulation was suppressed in GFAP-IκBα-dn mice compared to WT at 11 dpi but not at 20 dpi, the time point corresponding to the peak of EAE motor symptoms, where the two genotypes showed equal levels of TNF gene expression.

### Inhibition of astroglial NF-κB suppresses the expression of oxidative stress-related genes in the optic nerve following experimental ON

In an effort to investigate multiple pathways potentially involved in ON, we assessed the expression of oxidative stress-related genes. First, we measured inducible nitric oxide synthase (NOS2) and found, similarly to all other genes we analyzed, that NOS2 was upregulated in WT mice with a remarkable increase beginning at 11 dpi. This upregulation, however, was almost fully suppressed in GFAP-IκBα-dn mice (Figure 3).

**Figure 4:**
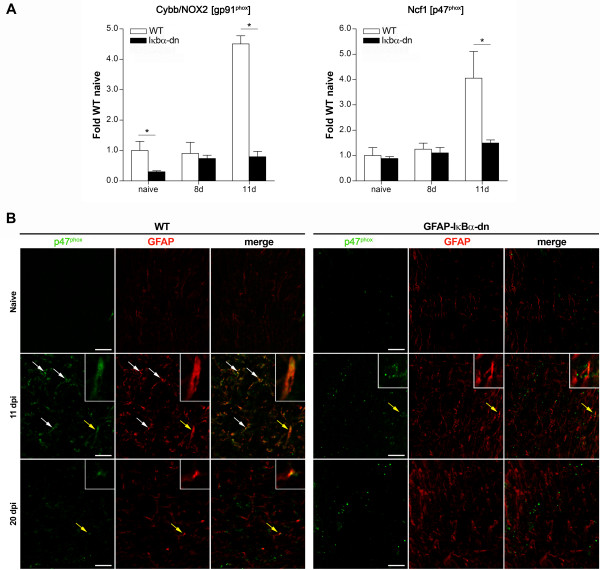
**Expression of the NAD(P)H oxidase subunits Cybb/NOX2 (gp91**^**phox**^**) and Ncf1 (p47**^**phox**^**) in the optic nerve of mice with ON. (A)** Gene expression was assessed in naïve animals and at 8 and 11 dpi. For each gene, results were expressed as fold of corresponding WT naive ± SEM after normalization to β-actin. Four mice/group/time were analyzed. *P < 0.05, one-way ANOVA, Tukey test. **(B)** p47^phox^ immunohistochemistry in the optic nerve of WT and GFAP-IκBα-dn mice in naïve conditions and at 11 and 20 dpi. Arrows point at astrocytes colocalizing with p47^phox^ protein. Yellow arrows identify the cells magnified in the inserts. Scale bar = 20 μm.

Second, we evaluated the expression of two key components of NAD(P)H oxidase, Cybb/NOX2 (encoding for gp91^phox^, the catalytic subunit of the complex) and Ncf1 (encoding for p47^phox^, the regulatory subunit of the complex). Excessive production of reactive oxygen species via NAD(P)H oxidase has been associated with neurological disease [24]. Recently, using the same GFAP-IκBα-dn mice employed in the present study, we demonstrated that NF-κB-regulated NAD(P)H oxidase in astrocytes is a crucial mediator of oxidative stress in a model of retinal ischemia and inhibition of its activity reduces damage by preventing RGC loss [25]. We found Cybb/NOX2 to be highly upregulated in WT mice at 11 dpi and this effect was fully abrogated in GFAP-IκBα-dn mice (Figure 4A). Interestingly, Cybb/NOX2 was significantly lower under naïve conditions in GFAP-IκBα-dn mice compared to WT, suggesting that transgenic mice may have an intrinsically reduced ability to generate ROS through NAD(P)H oxidase, hence a higher protection against oxidative stress. The upregulation of the Ncf1 subunit was also completely prevented in GFAP-IκBα-dn mice, further suggesting a reduced functionality of the NAD(P)H oxidase complex (Figure 4A). Consistently with the gene expression data, we found that p47^phox^ protein expression was upregulated in WT optic nerves compared to GFAP-IκBα-dn, with the highest level of expression at 11 dpi (Figure 4B). Double immuno-labeling with GFAP indicated that p47^phox^ is primarily localized to astrocytes, suggesting that astroglial NF-κB is a key transcriptional regulator of this molecule in the optic nerve.

Collectively, these data suggest that astrocyte may contribute to the early pathological events of ON through the production of oxidative species which, in parallel with other inflammatory mediators, could be causing myelin and axon damage.

### Inhibition of astroglial NF-κB significantly reduces RGC loss

Permanent damage of visual function in ON comes as a consequence of Wallerian degeneration of the optic nerve which ultimately leads to delayed death of RGCs [2,3]. Therefore, to assess the extent of irreversible visual damage, we evaluated RGC survival in WT and GFAP-IκBα-dn mice at various times after induction of EAE. Retinal whole mounts were immuno-labeled with the pan-neuronal marker NeuN and RGCs in the ganglion cell layer were counted.

**Figure 5:**
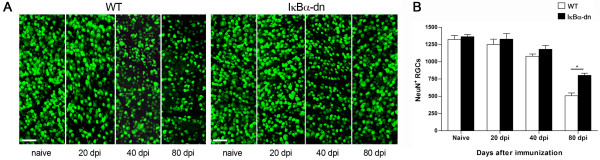
**Time-course of RGC loss in mice with ON. (A)** Immunohistochemistry with the pan-neuronal marker NeuN in flat-mounted retinas of WT and GFAP-IκBα-dn mice under naïve conditions and at various times after induction of EAE. **(B)** Quantification of NeuN-labeled RGCs with MetaMorph software. *P < 0.05, one-way ANOVA, Tukey test. Ten mice/group were analyzed. Scale bar = 50 μm.

In WT mice, we detected a minimal loss of RGCs at 20 dpi, which became significant only at 40 dpi, when compared to naïve mice. No significant loss of RGCs was detected in GFAP-IκBα-dn mice at these time points (Figure 5 A, B). A substantial loss of RGCs (about 60% of naïve) was detected in WT mice at the late chronic phase at 80 dpi, while the rate was significantly reduced (below 40%) in GFAP-IκBα-dn mice at corresponding time point (Figure 5B). This further underscores that astrocytes play a significant pathological role in the development of neurological deficits in ON, and blocking astrocyte-dependent detrimental cascades can almost fully protect from visual dysfunction associated with EAE.

## Discussion

This study provides evidence that astrocytes play a key role in the development of ON. This occurs through the activation of a transcriptional program regulated by NF-κB. Indeed, using a well characterized transgenic mouse model where NF-κB is specifically inactivated in astrocytes (GFAP-IκBα-dn mice), we show that NF-κB-dependent events initiated in astrocytes establish an environment that facilitates early axonal demyelination which takes place well ahead of leukocyte infiltration into the optic nerve. These data challenge the paradigm that ON is triggered by inflammatory events secondary to immune cell infiltration, and demonstrate that axonal demyelination in ON is, instead, an extremely early event driven by detrimental astrocyte-dependent effects. Our findings in ON are consistent with the pathological role that astrocytes play, as initiators of inflammation and oxidative stress, in many neurodegenerative diseases, as demonstrated by work of our and other laboratories [14,15,17-20,25-27].

### Activation of an NF-κB-regulated program in astrocytes is associated with early demyelination

Previous studies have reported axonal demyelination to coincide with blood-borne cell entry into the optic nerve parenchyma, suggesting that ON is triggered by immune-mediated events secondary to leukocyte infiltration [6,28,29]. Unexpectedly and in contrast with this largely accepted concept, our experiments in WT mice induced with EAE showed the appearance of significant myelin damage several days prior to the earliest signs of infiltration. We conducted a thorough time-course analysis of demyelination beginning as early as 5 dpi, and found that from 8 to 40 dpi, the numbers of myelinated axons were consistently and significantly reduced in WT mice. Conversely, GFAP-IκBα-dn mice were completely protected from axonal demyelination even at chronic time points after induction of EAE. The assessment of leukocyte infiltration conducted in parallel to the analysis of demyelination clearly underscored two major points: first, immune cell infiltration, as measured by CD45 immunohistochemistry, occurs after optic nerve demyelination reaches its peak (11 dpi); second, the extent and timing of immune cell infiltration is independent of astroglial NF-κB inactivation. In support of the latter, both WT and GFAP-IκBα-dn mice show similar profiles of CD45^+^ infiltrating cells at all time points evaluated. These data demonstrate that astroglial- rather than immune cell-dependent events are the key pathological determinants of the early stages of myelin loss in ON and challenge the established paradigm that infiltration of CD45^+^ cells is required for the onset of this pathology. Indeed, our data show that immune cells are virtually absent at the onset of ON, suggesting that most of the damage is facilitated by local rather than infiltrating cells. Nevertheless, we cannot exclude that low numbers of such sporadic infiltrating cells may still play a role at this stage. On the other hand, it is likely that leukocyte-dependent inflammation may contribute to optic nerve damage at later times, given the overwhelming presence of such cells in both genotypes at chronic disease (40 dpi). It is also worth pointing out that optic nerve demyelination is a pathological occurrence that precedes any manifestation of EAE-induced motor impairment, which initiates around day 10 and reaches peak around day 20 post induction (Figure 1A). This correlates with the human pathology where acute ON causing temporary visual impairment is often the earliest manifestation of MS, leading up to the initial diagnosis of the disease [1].

### Activation of NF-κB-dependent pathways in astrocytes sustains the inflammatory response in the optic nerve

Neuroinflammation and oxidative stress are two of the major culprits of CNS damage in MS and EAE [30-32]. Studies from our and other groups have shown that these processes are linked to the contribution of the astrocytes, either direct or indirect, and their ability to secrete toxic mediators which sustain and propagate CNS damage [14,26]. Indeed, using the same GFAP-IκBα-dn mice employed in this study, we previously showed that activation of NF-κB in astrocytes promotes the expression of pro-inflammatory cytokines and chemokines in the spinal cord of EAE-induced mice and suppression of this process via transgenic inhibition of astroglial NF-κB leads to functional recovery [14]. Similarly, in a mouse model of CNS-restricted NF-κB inactivation, van Loo and colleagues demonstrated that NF-κB inhibition prevented the expression of pro-inflammatory cytokines, chemokines, and the adhesion molecule VCAM-1 from astrocytes, suggesting that NF-κB-dependent gene expression in CNS resident cells creates a pro-inflammatory milieu that is critical for CNS inflammation and tissue damage in autoimmune demyelinating disease [26]. In agreement with these studies, our current work indicates that pathways downstream of astroglial NF-κB activation are key regulators/effectors of astroglia-induced neurotoxicity in ON as well. Specifically, we found that upregulation of IL1β, CXCL10, CCL5, and ICAM-1 was completely suppressed in the optic nerve of GFAP-IκBα-dn animals compared to WT. This is consistent with the fact that these molecules are massively produced by activated astrocytes and are transcriptionally regulated by NF-κB. The expression of TNF was more robust in WT versus GFAP-IκBα-dn at 11 dpi, but reached equal levels at 20 dpi, the time corresponding to the peak of EAE motor symptoms. This may be explained by the fact that, in contrast to the production of IL1β, CXCL10 and CCL5, microglia rather than astrocytes are the main cellular source of TNF in the injured CNS [14,33]. It is also important to notice that microglial cells are not affected by NF-κB functional inhibition in GFAP-IκBα-dn mice. Furthermore, TNF is highly produced by macrophages and T cells and this could be reflected in the peak of TNF expression at 20 dpi, when immune cell infiltration was found to be more abundant (Figure 2). Cell adhesion molecules are critical for immune cell activation and trafficking across the blood–brain barrier (BBB) into the CNS parenchyma [34]. We found that peak expression of ICAM-1, which occurred in WT mice at 11 dpi, was completely abrogated in GFAP-IκBα-dn mice (Figure 3). Because ICAM-1 is expressed by astrocytes and is one of the prototypical NF-κB-dependent genes [35,36], this may suggest that astrocytes can facilitate immune cell entry into the optic nerve, hence sustaining immune-inflammation occurring at the later stages of the disease (Figure 2).

It is important to emphasize that, albeit minimal, the first signs of demyelination were already noticeable as soon as 5 dpi (Figure 1B, C), the time point where massive changes in gene expression are not yet detectable. This suggests that early astrocyte-regulated signals are participating in the very initial stages of ON induction, acting in parallel to the above mentioned pro-inflammatory mediators and perhaps independently of *de novo* gene expression. Multiple injury mechanisms might be involved, including an increased production of nitric oxide (NO) or excitotoxic levels of glutamate, which can be produced by activated white-matter astrocytes. Both elevated NO and glutamate were shown to be responsible for reduced energy metabolism, and cause axonal damage [37]. Although a significant increase in expression of the *Nos2* gene was evident by 11 dpi, activity levels of the corresponding enzyme or extracellular glutamate concentrations were not assessed in this study. Based on the elevated levels of glutamate and NO in the normal appearing white matter of MS patients, one of the current hypotheses is that axonal mitochondrial energy failure may lead to axonal degeneration in MS [38,39]. These putative mechanisms are currently under investigation in the laboratory.

### Activation of NF-κB-dependent pathways in astrocytes sustains the oxidative stress response in the optic nerve

In an effort to investigate multiple neurotoxic pathways potentially involved in ON, we assessed the levels of NOS2 and superoxide producing phagocytic NAD(P)H oxidase. Activation of these enzymes is known to cause oxidative damage to myelin and axons through the production of nitric oxide and superoxide, which can further react to produce the strong oxidant peroxynitrite [40]. We found, similarly to all other genes we analyzed, that NOS2 and the NAD(P)H oxidase subunits Cybb/NOX2 and Ncf1 were upregulated in WT mice beginning at 11 dpi, but not in GFAP-IκBα-dn mice, suggesting that astrocyte may contribute to the early pathological events of ON through the production of reactive oxygen species. These results are consistent with our previous observations in a model of retinal ischemia-reperfusion where we showed that oxidative stress and neuronal loss in the retina depend on NF-κB-regulated activation of Cybb/NOX2 in astrocytes [25]. Other groups have also demonstrated that astrocyte-mediated oxidative stress is a key factor in optic nerve damage. Fitzgerald and colleagues have shown that oxidative stress spreading via the astrocytic network is an early event during secondary degeneration, and containment of this phenomenon is required in order to prevent further damage to the nerve [41].

### Astrocyte-mediated toxicity contributes to RGC loss

In MS/EAE-associated optic neuritis RGCs die retrogradely following axonal injury. Consistent with numerous reports that show axonal injury and demyelination to precede RGC death, our data also indicate that RGC loss is secondary to optic nerve damage. The fact that GFAP-IκBα-dn were significantly protected from RGC death compared to WT mice is a further demonstration that toxic pathways activated in astrocytes are the initial determinant in the cascade of events leading, ultimately, to permanent loss of visual function in optic neuritis. Because the protection from RGC loss was not complete, it is reasonable to believe that other mechanisms, in addition to astrocyte-mediated events, may be responsible for RGC death. One possibility is that leukocytes infiltrating the optic nerve at later times, as we show in our CD45 immunostaining, may produce inflammatory molecules that can cause axonal damage and consequently RGC death. These events are not prevented by blocking astrocytic NF-κB and could account for the lack of full RGC protection seen in GFAP-IκBα-dn mice.

## Conclusions

In conclusion, our results provide evidence that astrocytes play a key role in the development of optic neuritis secondary to MOG-induced EAE. Astrocyte-mediated neurotoxicity is dependent on activation of a transcriptional program regulated by NF-κB. Importantly, our data point out that axonal demyelination is already severe early, in the pre-clinical phase, when upregulation of pro-inflammatory and oxidative stress-related genes and accumulation of their products is not yet significant. These results suggest that pro-inflammatory and reactive oxygen species are not likely to serve as the initial triggers of demyelination, despite such molecules may greatly contribute to the damage during acute disease. Other NF-κB-dependent events activated in astrocytes at very early stages of the disease must be implicated in the initiation of ON and are the main focus of our ongoing investigations. Our studies underscore that interventions specifically targeting the NF-κB transcription factor in astroglia may be of therapeutic value in the treatment of optic neuritis associated with multiple sclerosis.

## Abbreviations

CNS: Central nervous system; EAE: Experimental autoimmune encephalomyelitis; MOG: Myelin oligodendrocyte glycoprotein; MS: Multiple sclerosis; NF-κB: Nuclear Factor kappa B; ON: Optic neuritis; RGC: Retinal ganglion cell; ROS: Reactive oxygen species.

## Competing interests

The authors declare the absence of any competing interests.

## Authors’ contributions

JRB, VIS, and RB conceived the study and participated in its design and coordination. RB and VIS analyzed the data, and drafted the manuscript. RB was responsible for induction of EAE and immunohistochemistry. GD carried out optic nerve and retina isolations, immunohistochemistry, and RGC quantification. DB carried out quantification of PPD-stained myelinated axons. DI carried out optic nerve and retina isolations, real-time PCR, and immunohistochemistry. All authors read and approved the final manuscript.
